# Rapid emergence of transmissible SARS-CoV-2 variants in mild community cases

**DOI:** 10.1128/spectrum.03634-23

**Published:** 2024-03-14

**Authors:** Michael A. Crone, Seran Hakki, Joe Fenn, Jie Zhou, Carolina Rosadas de Oliveira, Kieran J. Madon, Aleksandra Koycheva, Anjna Badhan, Jakob Jonnerby, Sean Nevin, Emily Conibear, Romain Derelle, Robert Varro, Constanta Luca, Shazaad Ahmad, Maria Zambon, Wendy S. Barclay, Jake Dunning, Paul S. Freemont, Graham P. Taylor, Ajit Lalvani

**Affiliations:** 1 Section of Structural and Synthetic Biology, Department of Infectious Disease, Imperial College London, London, United Kingdom; 2 UK Dementia Research Institute Centre for Care Research and Technology, Imperial College London, London, United Kingdom; 3 London Biofoundry, Imperial College Translation and Innovation Hub, London, United Kingdom; 4 NIHR Health Protection Research Unit in Respiratory Infections, National Heart and Lung Institute, Imperial College London, London, United Kingdom; 5 Section of Virology, Department of Infectious Disease, Imperial College London, London, United Kingdom; 6 Department of Virology, Manchester Medical Microbiology Partnership, Manchester Foundation Trust, Manchester Academic Health Sciences Centre, Manchester, United Kingdom; 7 UK Health Security Agency, London, United Kingdom; 8 NIHR Health Protection Research Unit in Emerging and Zoonotic Infections, University of Oxford, Oxford, United Kingdom; Emory University School of Medicine, Atlanta, Georgia, USA

**Keywords:** SARS-CoV-2, COVID-19, community, variants, immune escape, immunodeficiency

## LETTER

The emergence of viral mutations in severely immunodeficient patients with prolonged viral shedding is believed to be a significant source of highly infectious SARS-CoV-2 variants. We observed that community cases can also rapidly develop viruses with viable, culturable mutations with the potential for immune escape during the normal course of infection within 2 weeks of disease onset.

To fully capture the dynamics of variant emergence over the course of infection, a household contact study design with serial nasopharyngeal (NPS) swabbing was utilized to recruit incident delta variant COVID-19 cases (1). In 11 of 343 delta-exposed individuals, the viral growth phase was captured, and at least four consecutive NPS samples with the requisite concentration of RNA for whole-genome sequencing (WGS) (>1,000 RNA viral copies/m) were collected (Table S1). We performed WGS, viral culture plaque assays, and lateral flow devices (LFDs) serially on the viral transport media from the daily NPS samples as previously described (1) (see Supplemental Methods). Mutations were considered to be of biological significance if they were detected in >5% of sequencing reads, their proportion relative to all other reads changed over time, and if they previously been reported as conferring immune escape properties.

**Fig 1:**
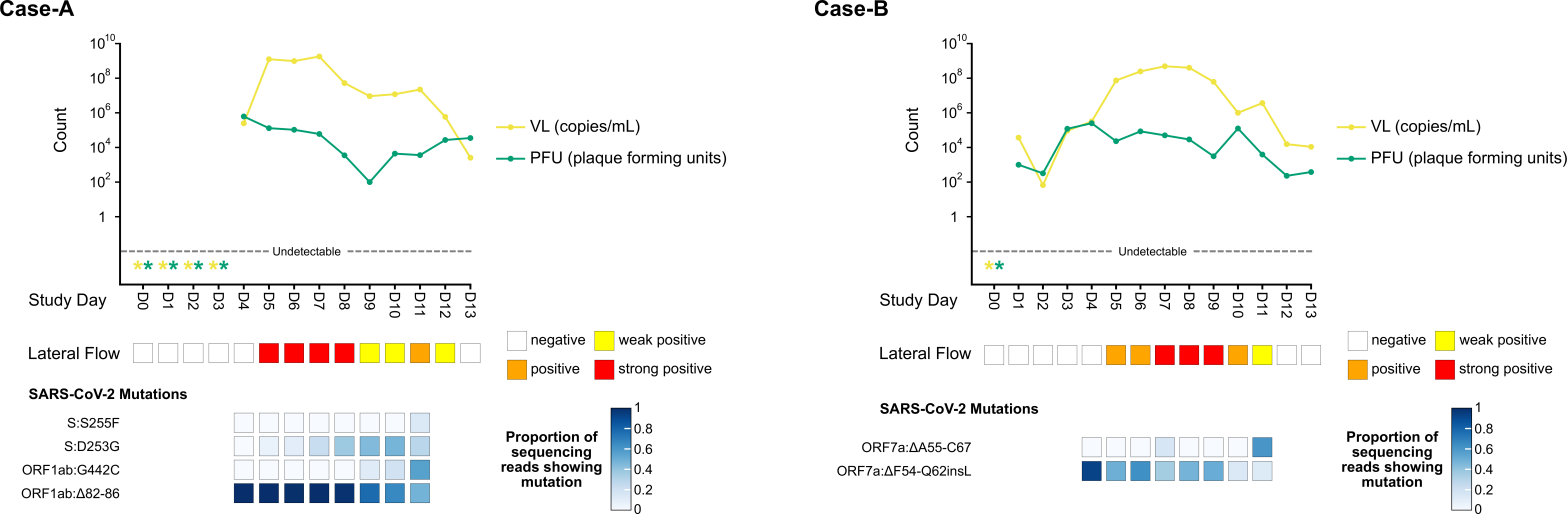
SARS-CoV-2 viral dynamics captured through daily sampling for Case A and Case B and a sequencing summary for nucleotide polymorphisms and indels in coding genes. Diagrams show the change in RT-qPCR viral load (VL) (yellow lines) and number of plaque-forming units (PFU) obtained using a plaque assay over the course of infection (green lines). Undetectable VL and PFU measurements are indicated by a star. Lateral flow results show the intensity of bands present on testing over the course of infection. Sequencing summarizes the results for any single-nucleotide polymorphisms and indels in coding genes that met all three of our significance criteria (the mutation needed to be found in at least 5% of the sequencing reads at a particular position, the mutant virus proportion needed to increase or decrease over time, and the mutation had to be within genes where its effect had been previously described or where mutations could play a role in immune escape based on gene function. All viral mutations for Case A and Case B can be visualized in Fig. S1 and S2, respectively.

Among the 11 cases, only Case A, who was vaccinated, showed significant mutations, including D253G and S255F nonsynonymous mutations in the Spike N-terminal domain (Fig. 1; Fig. S1). Of significance is that the mutations were found independently (on different reads) in the sequencing data, indicating that they developed independently during the latter stages of infection. These mutations negatively affect binding efficacy of anti-spike neutralizing antibodies (2, 3). The D253G mutation is associated with a variant of interest (4), while the S255F mutation has been detected in chronically infected individuals (5). The impact of vaccination on the development of these mutations is uncertain as the presence of pre-existing antibody responses to the Spike N-terminal domain prior to infection was unknown. These mutations temporally associated with viral rebound, suggesting that immune-escape mutations drove prolonged viral shedding rather than the extended shedding leading to the emergence of immune-escape variants (6). We have defined viral rebound as a significant increase (100-fold) in infectious virus on viral plaque assay (viral plaque assay results for Case A and Case B are found in Table S2). Of the remaining 10 cases, Case E had a neutral mutation (ORF1ab:T283I) (7), while Case B showed deletions in ORF7a (DF54-Q62insL and DA55-C67) (Fig. S2 and S3). These deletions observed in Case B hinder the inclusion of serine incorporator 5 (SERINC5) during virion budding, and studies have found that ORF7a deletion mutants maintain this inhibitory function (8).

Both Case A and Case B demonstrated an unusual persistence of infectious virus shedding, beyond the average duration of 5 days (IQR 3–7) (1) that extended beyond the sampling period (10 and 13 days of plaque assay positivity for Case A and Case B, respectively), despite negative LFDs in the later stages of infection (Fig. 1; Fig. S3). Both cases had type II diabetes and were overweight (BMI 27–28) but had good glycemic control, maintaining HbA1c levels below 48 mmol/mL for the 4-year pre-infection (Fig. S4) and had no history of immunosuppression or recurrent infections.

While our cohort size was small, our longitudinal WGS analysis provides novel evidence of potentially infectious immune-escape mutations originating within mild, self-resolving community-based patients rather than being transmitted from immunosuppressed patients. This process is likely to occur regardless of the dominating variant in circulation. Our findings, therefore, provide a crucial proof of concept that community cases of COVID-19 serve as an underestimated and potentially significant source of novel variants. These variants likely played a role in the emergence of successive highly infectious variants observed during the COVID-19 pandemic.

## Data Availability

All relevant assembled and raw sequence data have been deposited at the GISAID and NCBI under identifiers listed within the supplemental data.

## SUPPLEMENTAL MATERIAL

Tables S1-S3, Fig. S1-S3, additional experimental details spectrum.03634-23-s0001.docx Supplemental tables, figures, and methods.

ASM does not own the copyrights to Supplemental Material that may be linked to, or accessed through, an article. The authors have granted ASM a non-exclusive, world-wide license to publish the Supplemental Material files. Please contact the corresponding author directly for reuse.
